# Gut microbiota-metabolism-immunity axis: a novel perspective of traditional Chinese medicine in hepatocellular carcinoma treatment

**DOI:** 10.1186/s13020-026-01357-5

**Published:** 2026-03-24

**Authors:** Xuemei Zhang, Liming Zheng, Jia Shi, Shihan Yu, Wenlan Zheng, Hao Liu, Zhengzheng Wu, Hai Feng, Yueqiu Gao, Zhuo Yu

**Affiliations:** 1 Department of Hepatopathy, Shuguang Hospital Affiliated to Shanghai University of Traditional Chinese Medicine, 528 Zhangheng Road, Pudong New Area, Shanghai, 201203 China; 2 Institute of Infectious Disease, Shuguang Hospital Affiliated to Shanghai University of Traditional Chinese Medicine, Shanghai, 201203 China

**Keywords:** Traditional Chinese Medicine, Gut microbiota, Microbial metabolites, Immunity, Hepatocellular carcinoma

## Abstract

Hepatocellular carcinoma (HCC) is a highly aggressive malignancy characterized by complex metabolic dysregulation and immune evasion mechanisms, leading to poor prognosis and limited treatment efficacy. Growing evidence underscores the critical involvement of gut microbiota dysbiosis in HCC pathogenesis, wherein microbial communities and their bioactive metabolites dynamically influence the hepatic immune microenvironment and drive tumorigenesis. Emerging evidence indicates that Traditional Chinese Medicine (TCM) exerts therapeutic effects against HCC by modulating the gut-liver axis through multiple mechanisms, including remodeling gut microbial composition, regulating microbiota-derived metabolites, and fine-tuning hepatic immune responses, thus establishing a novel effective paradigm for HCC therapy. This article comprehensively reviews the intricate interactions between TCM and the gut microbiota-metabolism-immunity axis, elucidating the pivotal roles of gut microbiota and their metabolites in HCC pathogenesis and progression. Furthermore, we systematically summarize recent advances in understanding how TCM exerts anti-HCC effects through microbiota-mediated immunomodulation, with a particular focus on regulatory mechanisms involving microbial metabolites and hepatic immune responses. These findings provide valuable insights and establish a conceptual framework for future research in this burgeoning field.

**Graphical Abstract:**

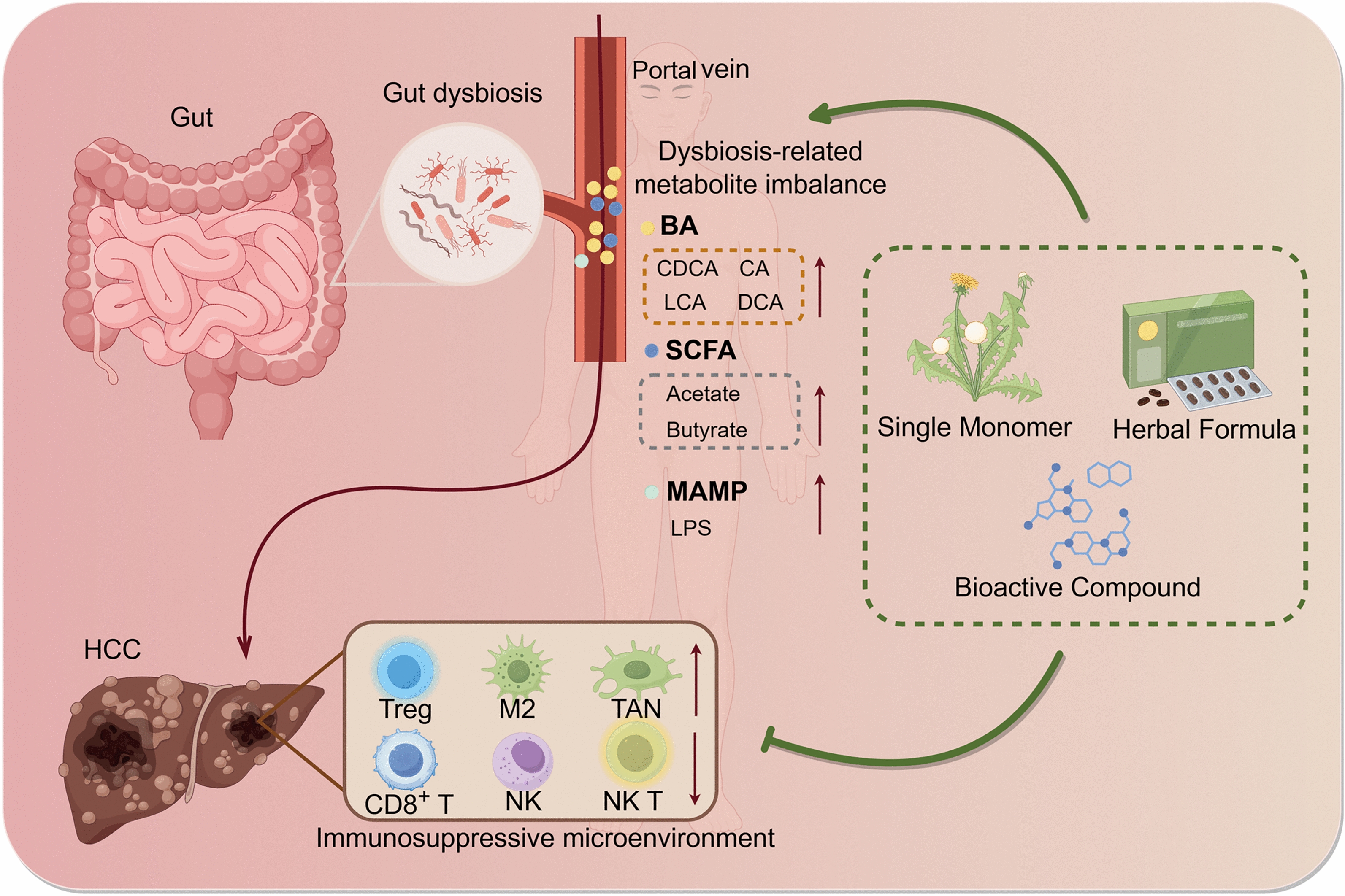

## Highlights

- The gut-liver axis is pivotal in HCC pathogenesis and therapy.
- Gut microbiota-derived metabolites critically regulate HCC immunity.
- Traditional Chinese Medicine ameliorates HCC via multitargeted modulation of the gut-liver-immune axis.
- Traditional Chinese Medicine reprograms tumor immunity via gut microbiota remodeling.
- Traditional Chinese Medicine offers a novel holistic paradigm for HCC management.

## Introduction

Hepatocellular carcinoma (HCC), accounting for approximately 90% of all primary liver malignancies, has emerged as the third leading cause of cancer-related deaths worldwide[1]. The development of HCC is primarily driven by well-defined etiological factors, such as chronic hepatitis B/C virus infection, alcohol abuse, and metabolic dysfunction-associated steatotic liver disease (MASLD, formerly known as non-alcoholic fatty liver disease, NAFLD)—particularly its inflammatory subtype, metabolic dysfunction-associated steatohepatitis (MASH, formerly known as non-alcoholic steatohepatitis, NASH) [2]. Therapeutic options for HCC are stratified and tailored according to disease stages: curative-intent surgical interventions, such as resection, transplantation, or local ablation, proves to be effective for early-stage patients, whereas approximately 70–80% of patients with advanced, inoperable disease require systemic therapies [3, 4]. Current first-line systemic therapies, such as multi-kinase inhibitors (e.g., sorafenib, lenvatinib) and immune checkpoint blockade (ICB), are plagued by substantial limitations. For instance, multi-kinase inhibitors often lead to the eventual emergence of drug resistance, while ICB exhibits a relatively low response rates (e.g., 15–20% for anti-PD-1 monotherapy) and is associated with severe immune-related adverse events in up to 30% of cases [5]. These therapeutic challenges highlight the critical need for further investigation into HCC pathogenesis and the development of mechanistically distinct treatment strategies.

Emerging evidence has demonstrated that the gut microbiota and its metabolites act as pivotal regulators of HCC pathogenesis and progression [6, 7]. Clinical studies reveal that HCC patients consistently demonstrate two hallmark intestinal alterations: significant gut microbial dysbiosis characterized by reduced microbial diversity, and intestinal barrier dysfunction with increased permeability [8]. Therapeutic modulation through probiotic administration in preclinical models has shown triple benefits: restoration of microbiota homeostasis, attenuation of hepatic inflammatory responses, and improvement of surgical outcomes [9, 10]. Notably, dietary cholesterol has been identified as a potent environmental trigger of gut microbiota dysbiosis and its metabolites disorder, constituting a critical point in the process from MASLD to HCC [11]. Mechanistically, cholesterol-driven microbiota alterations provoke a characteristic metabolites dysregulation marked by hepatic accumulation of primary bile acids (BAs) such as taurocholic acid (TCA), which exacerbates hepatic lipotoxicity and inflammatory infiltration [11]. It has also been identified that microbiota-derived secondary BAs like deoxycholic acid (DCA) function as immunomodulatory signals that modulate the natural killer T (NKT) cells to regulate antitumor immune surveillance [12]. These findings collectively underscore the gut-liver axis as a fundamental metabolic-immunological regulatory network in HCC development.

Traditional Chinese medicine (TCM) theory posits that HCC pathogenesis arises from a complex interplay of intrinsic Zhengqi (vital energy) deficiency, exogenous pathogenic factors invasion, and persistent blood stasis. TCM employs a holistic and personalized therapeutic approach for managing HCC. TCM interventions, encompassing herbal monomers, bioactive extracts, and classical compound formulas, exhibit unique advantages in oncology due to their multi-component nature and poly-pharmacological effects. Accumulating evidence demonstrates that TCM exerts anticancer effects by remodeling gut microbial ecosystems and regulating microbiota-derived metabolites, ultimately improving clinical outcomes in HCC patients [13, 14]. This review systematically investigates the physiological regulation of the gut-liver axis, the pathogenic mechanisms of microbiota-derived metabolites in HCC progression, and TCM-mediated modulation of gut-microbiota-immune crosstalk, providing translational perspectives for the development of microbiota-targeting HCC therapies.

## Gut-liver axis and microbiota

The bidirectional communication between the gut and liver constitutes a core regulatory network that governs hepatic physiology and pathology. Within this network, the gut microbiota has emerged as a pivotal driver, significantly influencing HCC pathogenesis. Intriguingly, the "Liver-Intestine Correlation" doctrine proposed millennia ago in TCM based on holistic visceral manifestation theory, exhibits a remarkable conceptual consistency with the modern "gut-liver axis" framework [15, 16]. This chapter will systematically dissect the structural components, physiological functions, and pathological dysregulation of the gut-liver axis. Furthermore, it will clarify the hierarchical relationships within microbiota-centered regulatory axes and elaborate on the functional roles of the gut microbiota in maintaining hepatic homeostasis, thereby laying a mechanistic foundation for understanding TCM-mediated HCC interventions.

### The differentiation and interconnections of gut microbiota-metabolism-immunity axis

The intricate interplay between gut microbiota, microbial metabolites, and hepatic immunity constitutes the pathological core of HCC progression, yet the hierarchical relationships within this network are often obscured by overlapping mechanisms. Based on validated preclinical and clinical evidence, we propose a "gut microbiota-metabolism-immunity" framework to delineate functional boundaries and interconnections. This framework integrates the "upstream trigger" (gut microbiota dysbiosis), "middle mediator" (microbial metabolite abnormalities), and "downstream effect" (hepatic immune microenvironment remodeling) into a continuous pathological cascade, representing a top-tier regulatory axis spanning HCC initiation, progression, and outcome. The essence of this axis lies in a vicious cycle: gut microbiota imbalance induces metabolic disorders, which further exacerbate immune suppression, collectively contributing to HCC development. Specifically, HCC patients exhibit a characteristic disruption of gut microbial structure, with enrichment of pro-inflammatory taxa (e.g., *Desulfovibrio*, *Escherichia coli*) and depletion of beneficial genera (e.g., *Bifidobacterium*, *Akkermansia muciniphila*) compared to healthy controls [17, 18]. This dysbiosis directly impairs the microbial substrate foundation required for normal metabolite synthesis. In line with the microbial imbalance, the level of secondary bile acids, such as deoxycholic acid (DCA) and lithocholic acid (LCA), is significantly increased in HCC patients compared to healthy individuals. Concurrently, short-chain fatty acids (SCFAs), such as acetate, butyrate, and indole-3-propionate (IPA) are decreased [19, 20]. These metabolites are transported into the liver via the portal vein, a process that is exacerbated by intestinal barrier dysfunction [21, 22]. Once in the liver, these metabolites directly modulate hepatic immune cell function. For instance, DCA inhibits the activity of Ca^2^⁺-ATPase (PMCA) in the plasma membrane of CD8⁺ T cells, leading to a 40% reduction in interferon-γ (IFN-γ) secretion [23], while lipopolysaccharide (LPS) activates the Toll-like receptor 4 (TLR4)/nuclear factor κB (NF-κB) pathway in Kupffer cells, which promotes M2 polarization[24]. These processes collectively contribute to the formation of the immunosuppressive microenvironment.

Clinically, the degree of imbalance within this master axis correlates significantly with HCC staging, as evidenced by a lower gut microbial Shannon diversity index in BCLC Stage C patients compared to Stage A patients, accompanied by elevated serum DCA and reduced IFN-γ [25, 26], supporting its potential as a marker of disease progression. Functionally, the "gut microbiota-metabolite" axis, acting as the "upstream-middle" connecting link, focuses on microbial enzyme-driven metabolite synthesis and transformation (e.g., *Bacteroides ovatus*-derived BSH mediating bile acid conversion, *Lactobacillus acidophilus*-derived acetate kinase regulating acetate production [27–29]). Complementarily, the "metabolite-immunity" axis, as the "middle-downstream" link, mediates hepatic immune regulation via metabolite-cell surface receptor-signaling interactions (e.g., LPS-TLR4 in Kupffer cells, DCA-PMCA in CD8⁺ T cells [23]), with mechanistic studies confirming their functional relevance in HCC models.

### Physiological and pathological mechanisms of the gut-liver axis

The gut-liver axis functions as a sophisticated bidirectional communication network mediated by the portal venous system, biliary excretion, and immunological crosstalk [30, 31]. Physiologically, the liver receives approximately 75% of hepatic blood flow via the portal vein, which transports gut-derived nutrients, microbial components, and metabolites. Within the liver, hepatic sinusoidal endothelial cells and Kupffer cells act synergistically to clear these substances; for example, Kupffer cells phagocytose 90% of bacteria delivered via the portal vein, contributing to the maintenance of immune tolerance [15, 16]. Similarly, bile acid homeostasis is also maintained through this axis: hepatocytes synthesize primary bile acids (e.g., cholic acid (CA), chenodeoxycholic acid (CDCA)), which are secreted into the intestine, where the gut microbiota converts them into secondary bile acids (e.g., DCA, LCA). Ninety-five percent of these secondary bile acids are then reabsorbed via the ileal bile acid transporter (IBAT), thus completing the enterohepatic circulation [32, 33]. Furthermore, gut-associated lymphoid tissue (GALT) serves as the first line of defense against pathogens, with the intestinal barrier confining the translocation of pathogen-associated molecular patterns (PAMPs; e.g., LPS) into the systemic circulation, thereby preserving hepatic immune equilibrium[21, 34].

In HCC, this axis undergoes pathological dysregulation, HCC-related factors, such as chronic inflammation and alcohol-induced injury, can reduce the expression of tight junction proteins (zonula occludens-1 (ZO-1), occludin) and mucin-2 (MUC-2), leading to impaired intestinal barriers and increased translocation of PAMP/metabolite [22, 35]; A growing body of research has also confirmed that intestinal-derived LPS enters the liver through the impaired intestinal barrier and promotes the occurrence and development of HCC by activating the TLR4 signaling pathway, which can drive tumor cell proliferation, induce chronic hepatic inflammation and oxidative stress, remodel the tumor microenvironment, maintain cancer stem cell stemness [24, 36–38]. TLR4 is highly expressed in HCC tissues and associated with poor prognosis [36], and strategies such as gut microbiota modulation and pathway inhibition targeting the LPS/TLR4 axis have provided novel directions for the prevention and treatment of HCC. It is important to note that intestinal barrier dysfunction, elevated LPS levels, and gut microbiota dysbiosis are also frequently observed in cirrhosis, thus the observed alterations in HCC patients may partially stem from a pre-existing cirrhotic background rather than being exclusively HCC-specific [39–41]. In addition, gut microbiota dysbiosis alters bile acid metabolism which also drives the occurrence and development of HCC, specially, *Bacteroides*-driven DCA elevation, thereby aggravating hepatic inflammation through DCA-induced senescence-associated secretory phenotype (SASP) in hepatic stellate cell (HSC) [42–44]. Pathological dysregulation of the gut-liver axis not only reveals the intrinsic association between intestinal and hepatic pathological changes, but also provides novel perspectives and theoretical foundations for the prevention and treatment of HCC via targeting the LPS/TLR4 axis, gut microbiota and bile acid metabolism.

### Composition, functions, and dysregulation of the gut microbiota

The gut microbiota, a complex ecosystem comprising trillions of commensal microorganisms with a collective genetic repertoire approximately 150 times larger than the human genome, plays a crucial role in maintaining hepatic health [45]. Phylogenetic analysis has identified seven dominant phyla within this ecosystem: Firmicutes, Bacteroidetes, Actinobacteria, Fusobacteria, Proteobacteria, Verrucomicrobia, and Cyanobacteria [46]. Among these, Firmicutes and Bacteroidetes constitute over 90% of the total abundance, and their ratio (F/B ratio) serves as a key marker of gut homeostasis [47]. Critical beneficial genera include *Akkermansia muciniphila*, a mucin-degrading bacterium that enhances intestinal barrier function via MUC-2 upregulation and LPS reduction [34, 48], and *Bifidobacterium/Lactobacillus*, which produce SCFA, inhibit pro-inflammatory cytokines and regulate T cell immunity [49, 50]. Under homeostatic conditions, the gut microbiota contributes to host health through various mechanisms, including fermenting dietary fiber to produce SCFAs, which serve as energy sources for colonocytes and acts as metabolic regulators [26, 58]), stimulating the expression of tight junction protein mucin, as well as secreting antimicrobial peptides to prevent pathogen colonization [18, 39], regulating GALT function, such as butyrate promoting regulatory T cell (Treg) differentiation and primary bile acids facilitating natural killer T cell (NKT) recruitment [11, 59]), and metabolizing drugs/toxins, including bile acid conversion and dietary carcinogen detoxification [26, 45]. In HCC, dysbiosis is observed, characterized by a significantly increased F/B ratio [28, 31], enrichment of pro-inflammatory taxa (e.g., *Desulfovibrio*, *E. coli*) and depletion of beneficial genus (e.g., *Bifidobacterium, Akkermansia*) [31, 37]. This dysbiosis can promote HCC development through multiple pathways: Pro-inflammatory bacteria produce hydrogen sulfide and LPS, activating TLR4/NF-κB signaling and driving chronic hepatic inflammation [28, 42]; reduced SCFA production and increased secondary bile acids disrupt lipid metabolism, promoting hepatocyte steatosis, a key precursor to HCC [53, 65]; and microbial metabolites, such as iso-LCA, can inhibit NK cell/CD8⁺ T cell function, fostering an immunosuppressive microenvironment [55, 56]. Notably, probiotic supplementation with species such as *A. muciniphila* and *Lactobacillus bifidus* has shown promise in restoring microbial homeostasis, reducing hepatic inflammation, and improves surgical outcomes in immunocompetent HCC preclinical models and clinical trials [9, 82], highlighting the therapeutic potential of microbiota modulation.

## Dysregulation of the gut microbiota-metabolism-immunity axis in HCC

### Gut dysbiosis in HCC

Accumulating evidence, both preclinical and clinical, has established a strong association between gut microbial dysbiosis and HCC development (Table 1). Clinical investigations reveal significant gut microbiota dysbiosis in HCC patients compared to healthy controls [51], characterized by a marked increase in potentially pathogenic microorganisms, including *Escherichia coli* and members of the phylum Actinobacteria, alongside a substantial reduction in beneficial genera such as *Faecalibacterium, Ruminococcus, Bifidobacterium, Lactobacillus, and Ruminoclostridium* [18, 25, 26, 52–55]. These findings are corroborated by observations in diverse murine models of hepatocarcinogenesis. For instance, streptozotocin-high fat diet (STZ-HFD)-induced MALSD-HCC murine models exhibit significant alterations in microbial composition, notably the enrichment of pro-inflammatory taxa such as *Atopobium spp.*, *Bacteroides acidifaciens*, *Bacteroides spp.*, *Bacteroides uniformis*, *Bacteroides vulgatus*, *Clostridium xylanolyticum*, *Clostridium cocleatum*, and *Desulfovibrio spp* [17]. Similarly, diethylnitrosamine (DEN)-induced hepatocarcinogenesis models demonstrate significant increases in *Escherichia coli*, *Atopobium*, *Coriobacterium*, *Collinsella*, and *Eggerthella*, concomitant with a decrease in beneficial genera including Bifidobacterium, Enterococcus, and Lactobacillus [56]. Furthermore, hypercholesterolemia-associated MASLD-HCC progression is characterized by a distinct dysbiosis signature, featuring an overgrowth of mucin-degrading *Mucispirillum* and endotoxin-producing Desulfovibrionaceae, along with a reduction in *Bacteroides* and *Bifidobacterium* populations [11]. These reproducible microbial signatures across diverse HCC models underscore the conserved role of gut microbiota in hepatocarcinogenesis. Elucidating the mechanistic basis of the gut microbiota-HCC axis holds the potential to not only advance our understanding of novel hepatocarcinogenic pathways, but also to pioneer innovative microbiota-modulating therapeutic strategies, such as probiotic intervention and fecal microbiota transplantation. Future research should prioritize establishing correlations between specific microbial alterations and (i) HCC molecular subtypes; (ii) clinical prognosis, and (iii) treatment responsiveness, to facilitate the transition of gut microbiome modulation into precision medicine paradigms.

**Table 1:** Alterations in gut microbiota composition in HCC animal models and clinical patients

Preclinical research		
Condition	Microbes associated	Refs
STZ-HFD induced MASH-HCC	↑*Atopobium* spp., *Bacteroides* spp., *Bacteroides vulgatus*, *B. acidifaciens*, *B. uniformis*, *Clostridium cocleatum*, *C. xylanolyticum*, *Desulfovibrio* spp.	[17]
DEN induced HCC	↑*Escherichia coli, Atopobium cluster, Atopobium, Collinsella, Eggerthella, Coriobacterium*, ↓*Lactobacillus species, Bifidobacterium species, Enterococcus species*	[56]
HFHC induced MASLD-HCC	↑*Mucispirillum*, *Desulfovibrio*, *Anaerotruncus*, *Desulfovibrionaceae*, ↓*Bifidobacterium*, *Bacteroides*	[11]
DMBA-HFD induced HCC	Altered gut microbiota	[43]
MYC transgenic spontaneous HCC	*↑*Gram-positive bacteria*,* Bacteria mediating primary-to-secondary bile acid conversion*, Clostridium scindens*	[12]
DMBA or DMBA-HFD induced HCC	*↑*Gram-positive bacteria	[57]

Clinical research		
HCC	*↑Escherichia coli*	[52]
Primary HCC	↑*Proteobacteria, Desulfococcus, Enterobacter, Prevotella, Veillonella* ↓*Cetobacterium*	[25]
Primary HCC	↑*Enterobacter ludwigii, Enterococcaceae, Lactobacillales, Bacilli, Gammaproteobacteria, Veillonella*, ↓diversity of *firmicutes, firmicutes/bacteroidetes, Clostridia, Subdoligranulum*	[54]
MASLD HCC	↑*Bacteroides* ↓*Akkermansia, Bifidobacterium*	[26]
Liver cirrhosis-induced HCC	↑*Neisseria*, *Enterobacteriaceae*, *Veillonella*, *Limnobacter*, ↓*Enterococcus*, *Phyllobacterium*, *Clostridium*, *Ruminococcus*, *Coprococcus*	[40]
MASLD related HCC	Gut microbial α-diversity ↓, ↑*Proteobacteria, Enterobacteriaceae, Bacteroides xylanisolvens, B. caecimuris, Ruminococcus gnavus, Clostridium bolteae, Veillonella parvula*, *↓Oscillospiraceae, Erysipelotrichaceae*	[51]
Hepatitis B related HCC	↑*Escherichia coli, Shigella, Enterococcus* *↓Faecalibacterium*, *Ruminococcus*, *Ruminoclostridium*	[53]
Early HCC	↑ *Actinobacteria,* Klebsiella *and *Haemophilus (producing LPS) ↓*Ruminococcus, Oscillibacter, Faecalibacterium*, *Alistipes*, *Phascolarctobacterium*, *Clostridium IV, Coprococcus* (butyrate-producing bacteria families)	[41]

### Gut barrier dysfunction in HCC

The intestinal barrier is a sophisticated, multi-layered defense system that maintains selective permeability, allowing for nutrients absorption while preventing the translocation of pathogenic microorganisms and harmful substances. This precisely regulated barrier system comprises four structurally and functionally integrated components[35]: (i) the mechanical barrier, formed by intestinal epithelial cells and their intercellular tight junction complexes (e.g., occludin and claudin family proteins); (ii) the chemical barrier, consisting of the mucus layer (particularly MUC-2) and antimicrobial peptides; (iii) the immune barrier, mediated by gut-associated lymphoid tissue (GALT); and (iv) the biological barrier, established by commensal microbiota[58]. Under physiological conditions, this intact barrier system strictly confines PAMPs, thereby preserving immune homeostasis in both the gastrointestinal tract and liver [34]. However, in HCC, disruption of intestinal barrier integrity, clinically termed "leaky gut", facilitates the translocation of bacterial components, including LPS, into the portal circulation [22]. Clinically, a progressive increase in serum LPS levels parallels the advancement of liver injury and HCC progression [41]. Furthermore, clinical investigations have demonstrated that elevated serum levels of tight junction proteins (e.g., ZO-1) correlate significantly with impaired barrier function, enhanced systemic inflammation, and advanced disease severity in HCC patients [59]. At the molecular level, LPS activates Toll-like receptor 4 (TLR4) signaling, initiating the nuclear factor kappa-B (NF-κB) pathway. This cascade promotes hepatic stellate cell (HSC) activation and subsequent secretion of pro-inflammatory cytokines (e.g., tumor necrosis factor-alpha (TNF-α) and interleukin-6 (IL-6)) and anti-apoptotic proteins, while simultaneously suppressing pro-apoptotic factors such as caspase-3. These molecular alterations collectively drive aberrant hepatocyte proliferation and apoptosis resistance, ultimately facilitating HCC development and progression [24, 37]. Notably, antibiotic treatment in experimental HCC models has been shown to reduce tumor burden and prevent HCC progression, likely through depletion of gut microbiota and subsequent reduction of PAMPs [12]. Current research primarily focuses on the mechanical barrier maintained by epithelial tight junctions and the mucus barrier predominantly composed of MUC-2 as critical components of intestinal barrier function. These findings suggest that intestinal barrier function markers may serve as valuable indicators for monitoring HCC progression, and the TLR4/NF-κB signaling pathway represents a potential therapeutic target, offering anti-HCC benefits through selective modulation of gut microbiota-derived PAMPs via antibiotic intervention. Future research should focus on developing precision therapeutic strategies targeting the gut microbiota-barrier-liver axis, which may lead to novel approaches for HCC prevention and targeted treatment.

### Dysregulation of gut microbiota-derived metabolites in HCC

**Fig. 1:**
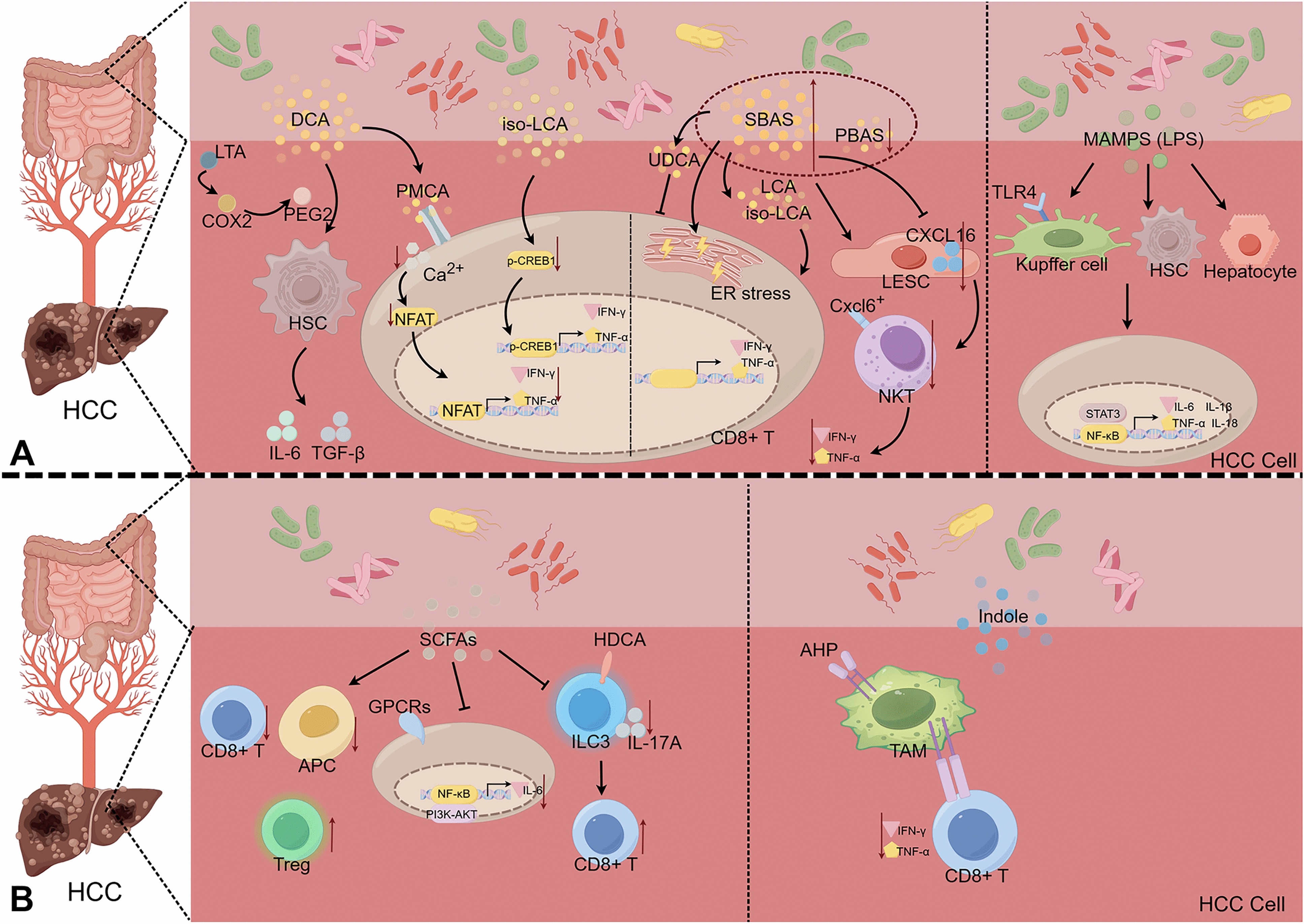
Disrupted Gut Microbiota-Metabolism-Immune Axis in HCC. Gut dysbiosis results in the aberrant production of various metabolites and microbial components, including secondary bile acids (e.g., DCA, iso-LCA), short-chain fatty acids (SCFAs), indole derivatives and microbial-associated molecular patterns (MAMPs), such as LPS and LTA. Consequently, these factors compromise intestinal barrier function, leading to their translocation to the liver. Within the liver, they modulate hepatic immune cells, including HSC, KCs, CD8⁺ T cells, NK cells, and TAMs, as well as signaling pathways such as NFAT, CREB1, TLRs, and NF-κB

Emerging evidence indicates that gut dysbiosis may facilitate the progression of HCC by altering microbial metabolite profile with increased production of harmful metabolites and a reduction in beneficial compounds [19]. These microbiota-derived metabolites can translocate to the liver via the gut-liver axis, influencing the hepatic immune microenvironment and acting as critical regulators in HCC pathogenesis (Fig. 1). Notably, bile acids and short-chain fatty acids have been identified as key mediators in this process.

#### Bile acids in HCC

Bile acids, catabolic products of cholesterol metabolism, play a pivotal role in the regulation of lipid, glucose, and energy homeostasis. Primary bile acids are transformed by gut microbiota into secondary bile acids, including deoxycholic acid (DCA), lithocholic acid (LCA), and ursodeoxycholic acid (UDCA) [5, 60]. Gut microbiota-derived secondary bile acids contribute to HCC progression through intricate crosstalk involving inflammatory and immune pathways [33]. Bile acids also play a critical role in modulating antitumor immunity. Specifically, microbiota-mediated primary bile acids enhance liver antitumor immunity by promoting chemokine-dependent accumulation of natural killer T (NKT) cells [12]. Primary bile acids upregulate chemokine CXCL16 expression in liver sinusoidal endothelial cells (LSECs), whereas microbial-derived secondary bile acids downregulate CXCL16, impairing NKT cell function [12]. However, isolithocholic acid (iso-LCA), produced by *Bacteroides ovatus*, suppresses NK cell cytotoxicity by reducing the production of interferon-γ (IFN-γ), tumor necrosis factor-α (TNF-α), perforin, and granzyme B, contributing to an immunosuppressive microenvironment [27]. Furthermore, DCA attenuates CD8⁺ T cell responses by inhibiting the Ca^2^⁺- nuclear factor of activated T cells (NFAT) signaling pathway through targeting the plasma membrane Ca^2^⁺ ATPase (PMCA) [23]. Recent studies have elucidated that aberrant accumulation of primary and secondary bile acids impairs CD8⁺ T cell function through distinct mechanisms: primary bile acids induce oxidative stress, while secondary bile acids trigger endoplasmic reticulum stress, collectively suppressing the secretion of IFN-γ, TNF-α, perforin, and granzyme B [61]. Clinical cohort studies have revealed that reduced ratios of secondary to primary bile acids are significantly associated with an increased risk of HCC [62]. Accumulating evidence indicates that LCA promotes HCC development by enhancing tumor cell proliferation, invasion, migration, epithelial-mesenchymal transition, and angiogenesis [63–66]. In contrast, UDCA exerts hepatoprotective effects by inducing cell cycle arrest and apoptosis, thereby suppressing tumorigenesis [42]. In obese murine models of HCC, gut dysbiosis leads to elevated accumulation of DCA in the liver and intestine, exacerbating senescence-associated secretory phenotype (SASP) in HSC and facilitating chemical carcinogen-induced HCC development [43]. Mechanistically, obesity-induced enrichment of Gram-positive gut bacteria results in the translocation of lipoteichoic acid to the liver, fostering a pro-carcinogenic microenvironment. Lipoteichoic acid acts synergistically with DCA to amplify SASP in HSC via TLR2-mediated upregulation of SASP factors and cyclooxygenase-2 (COX-2) expression, thereby accelerating hepatocarcinogenesis [57]. As critical metabolic hubs connecting gut microbiota with hepatic pathophysiology, disturbances in bile acids metabolism represent fundamental drivers of HCC pathogenesis. Consequently, multidimensional strategies aimed at rebalancing bile acids profiles, precisely modulating bile acids-related signaling pathways, and implementing holistic microbiota interventions may help overcome current therapeutic limitations in HCC management. Such approaches could pave the way for innovative treatment paradigms centered on the "microbiota-metabolism-immunity" axis.

#### Short-chain fatty acids in HCC

Short-chain fatty acids (SCFAs), including acetate, butyrate, and propionate, are small molecular metabolites produced by microbial fermentation of dietary fibers [20]. Functioning as key effector molecules of gut microbiota, SCFAs influence HCC pathogenesis through an integrated metabolic-immuno-epigenetic regulatory network. These compounds exert pleiotropic effects on epithelial barrier function and systemic immunity, primarily via modulation of G protein-coupled receptor signaling and histone deacetylase (HDAC) activity [67]. At the molecular level, acetate can exhibit antitumor immunomodulatory properties in HCC through HDAC inhibition-mediated suppression of IL-17A production by group 3 innate lymphoid cells [29], while butyrate supplementation significantly attenuates HCC proliferation and metastasis, mechanistically linked to calcium signaling pathway activation, subsequent disruption of intracellular calcium homeostasis, and reactive oxygen species generation [68]. Furthermore, probiotic strains such as *Lactobacillus acidophilus* exert chemopreventive effects in diet-induced MASLD-HCC models through SCFA-mediated downregulation of oncogenic signaling pathways [49, 50]. Clinical cohort studies have demonstrated that elevated SCFA concentrations correlate significantly with prolonged progression-free survival and overall survival in HCC patients [28]. Notably, SCFAs demonstrate context-dependent duality in HCC modulation [29, 69]. Recent animal studies reveal that high-fiber diets under conditions of gut dysbiosis paradoxically promote hepatocyte proliferation, fibrosis, and HCC development via elevated butyrate production [69]. Similarly, MASLD-HCC progression associates with increased butyrate and acetate levels that foster an immunosuppressive microenvironment characterized by regulatory T cell (Treg) expansion and concomitant reduction of CD8^+^ T cells and antigen-presenting cells (APCs) [51]. These paradoxical observations suggest that SCFAs effects are critically dependent on both intervention modality and concentration thresholds. Therapeutic targeting of SCFA metabolic pathways not only provides novel perspectives for HCC prevention but also demonstrates promising synergistic potential with existing treatment modalities. Future research should focus on elucidating cell type-specific mechanisms of SCFA action, developing precision modulation strategies, and establishing complete translational pipelines from microbial metabolites to clinically actionable targets. This comprehensive approach will facilitate the realization of SCFA-based interventions in HCC management.

#### Other microbiota-derived metabolites in HCC

The gut microbiota also significantly influences HCC regulation through modulation of indole metabolism. This pathway primarily depends on tryptophanase-expressing gut bacteria, including Clostridium, Bacteroides, Escherichia, Bifidobacterium, and Lactobacillus genera, which convert dietary tryptophan into indole derivatives such as indole-3-aldehyde, indole-3-acetic acid, indole-3-propionic acid, and indole-3-carboxylic acid [70, 71]. Several studies have highlighted the complex and sometimes opposing roles of these metabolites in HCC development. For instance, untargeted metabolomics analyses of HCC patient samples (portal, central venous serum, liver tissue, and feces) reveal significant elevations of DL-3-phenyllactic acid, L-tryptophan, glycocholic acid, and 1-methylnicotinamide in portal serum and HCC tissues, with these alterations correlating with impaired liver function and poor survival [72]. Conversely, reduced linoleic acid and phenol levels in portal blood and fecal samples may represent protective metabolic signatures against HCC [72]. Specific indole metabolites, like indole-3-propionic acid and indole-3-acetic acid, have demonstrated protective effects by maintaining intestinal barrier integrity, reducing bacterial translocation, and preventing microbial component leakage to the liver, thereby suppressing hepatic inflammation [73–76]. However, other indole derivatives can promote HCC; experimental studies in high-fat or high-cholesterol diet-induced spontaneous MASLD-HCC mouse models exhibit reduced levels of gut microbiota-derived 3-indolepropionic acid, which promotes MASLD-HCC progression by dysregulating hepatocyte lipid metabolism [11]. Consistently, MASLD-HCC patients show elevated fecal kynurenine and kynurenic acid levels alongside decreased tryptophan and indole-3-carboxylate concentrations [51]. Furthermore, microbiota-produced indole compounds can activate the aryl hydrocarbon receptor (AHR) on tumor-associated macrophages (TAMs), inducing macrophage polarization that suppresses CD8^+^ T cell activation and reduces IFN-γ and TNF-α production [77]. Additionally, indole-3-pyruvate has been shown to promote MYC-dependent hepatocarcinogenesis [78].

These findings collectively suggest that restoring metabolic homeostasis through strategies such as probiotic modulation, dietary fiber supplementation, or targeted intervention of metabolite receptors may offer novel avenues for HCC prevention and treatment. Future investigations should prioritize elucidating the precise regulatory mechanisms of specific metabolites and characterizing their synergistic effects with existing therapies. Such research will provide the theoretical basis for developing personalized therapeutic approaches targeting the "gut microbiota-metabolite-liver" axis.

## Regulation of gut microbiota-metabolism-immunity axis by TCM in HCC

**Fig. 2:**
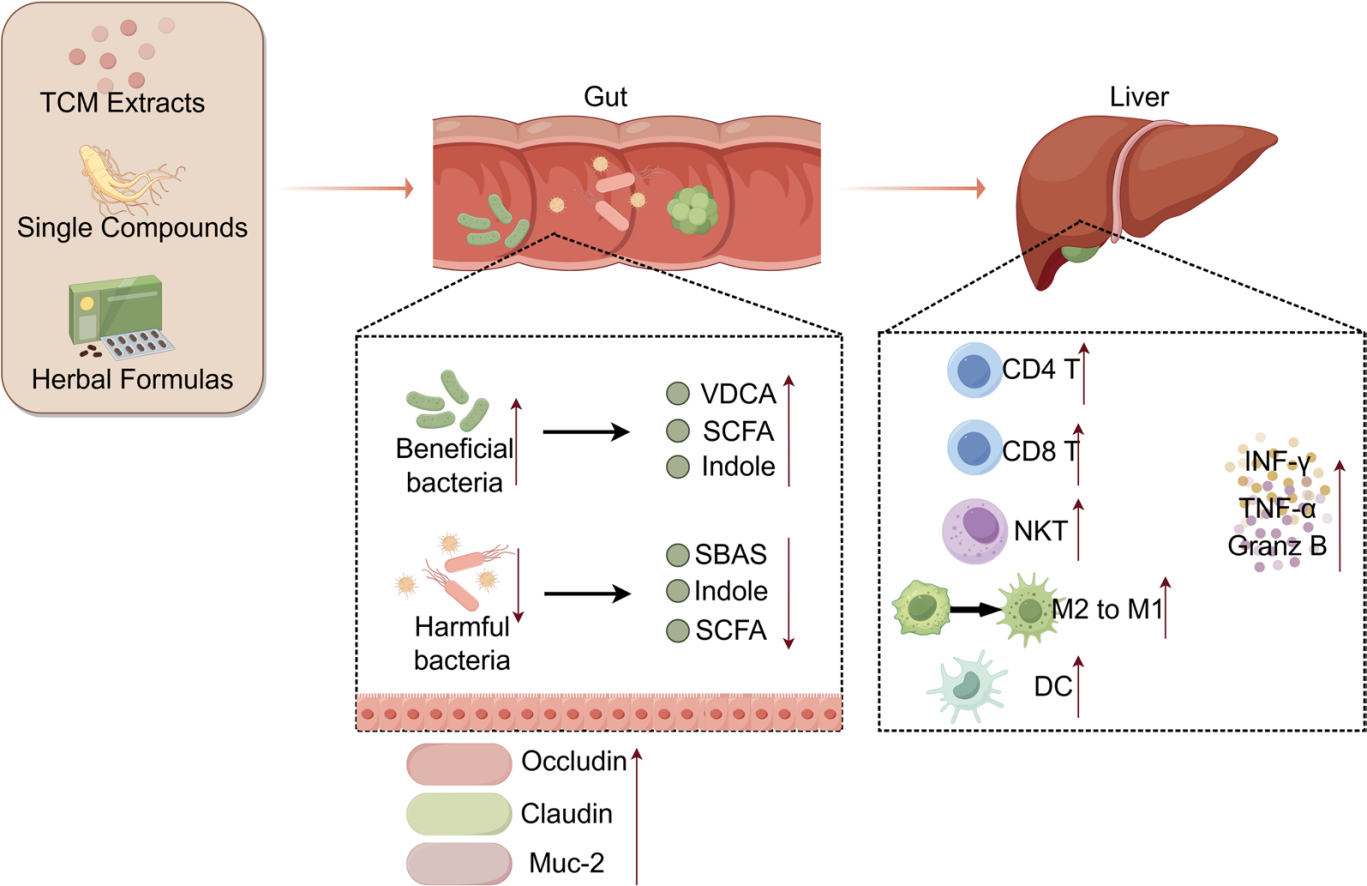
Regulation of Gut-Liver Immune Axis by Active Components of TCM. When acting on the intestine, TCM components (including extracts, single compounds, and compound formulations) bidirectionally modulate microbial balance by promoting beneficial bacteria and/or suppressing harmful bacteria. Consequently, TCM components regulate the levels of gut microbiota-derived metabolites, such as bile acids, short-chain fatty acids (SCFAs), and indole. Furthermore, they enhance intestinal barrier function through the upregulation of tight junction proteins (e.g., occludin, claudin) and MUC-2. These intestinal signals are then transmitted to the liver, modulating hepatic immune responses. Specifically, TCM components promote the proliferation of CD4⁺T, CD8⁺T, NKT cells, and dendritic cells (DCs), while also driving macrophage polarization from the M2 phenotype toward the pro-inflammatory M1 type. These immunological shifts are accompanied by increased secretion of immune-promoting cytokines, including interferon-gamma (IFN-γ), tumor necrosis factor-alpha (TNF-α), and granzyme B

TCM inhibits HCC by targeting multiple levels of the "gut microbiota-microbial metabolites-hepatic immunity" cascade (Table 2). TCM re-establishes gut-liver axis homeostasis, thereby counteracting the pathological interactions that drive HCC progression (Fig. 2).

**Table 2:** Traditional Chinese Medicine exerts anti-HCC effects by modulating the gut microbiota–gut microbiota metabolites–liver axis

Components	Research model/ Immune status	Changes of gut microbiota composition	Changes of gut microbiota metabolites	mechanism	Reference
Panax ginseng	HCC model established by HepG2 cell /immunodeficient model	*↑Coprococcus, Dehalobacterium, Roseburia, Anaerotruncus; ↓Bacteroides, Prevotella, Arthromitus, Gemmiger*	↑SCFAs and secondary BAs biosynthesis	Inhibiting chronic inflammatory response	[121]
Jiawei Xiaoyao San	DEN induced + chronic unpredictable mild stress/ immunocompetent model	*↑Firmicutes, Lachnospiraceae; ↓Bacteroidetes, Proteobacteria, Porphyromonadaceae unclassified, Bacteroides, Oscillibacter, *etc	Influencing differential metabolites and signaling pathways	anti-inflammatory, promoting immunomodulatory effect	[96]
Berberine	Orthotopic HCC model established by HepG2 cell-derived xenografts /immunocompetent model	*↑*butyric acid-producing intestinal flora	↑butyric acid	activating PPARδ to trigger apoptotic death	[79]
Huaier polysaccharides	Hepa1-6 inoculated into the armpits of C57BL/6 J mice /immunocompetent model	*↓Parasutterella; ↑Akkermansia*	↓the content of bile acids	inhibiting the polarization of M2 macrophages in tumor microenvironment and increasing the secretion of pro-inflammatory factors	[101]
Chlorogenic acids	Rat HCC model established by DEN/immunocompetent model	*↓Ruminococcaceae UCG-004;* *↑Lachnospiraceae incertae sedis,Prevotella 9*	Influencing differential metabolites related to microbiota	NA	[83]
Xiayuxue Decoction	HCC mouse model by DEN and CCL4/immunocompetent model	*↑ Bacteroides and Lactobacillus; ↓ Eubacterium*	↑ the content of primary bile acids	triggering NKT cells in the liver to produce interferon-γ to exert anti-HCC immune effects	[108]
Safflower yellow	DEN-induced HCC/immunocompetent model	*↓Alloprevotella,Ruminococcus, Barnesiella, Bacteroides, Ersipelotrichaceae incertae sedis*	NA	promoting CD8^+^ T-cell and Gr-1^+^ macrophage mediated immune suppression, decreasing TNF-α and NF-κB-mediated inflammation, regulating tumor immune microenvironment	[107]
quercetin	orthotopic HCC model established by hepa1-6/immunocompetent model	*↑Firmicutes, Actinobacteria, Verrucomicrobiota, Dubosiella and Akkermansia;* *↓ Lactobacillus and Lachnospira*	NA	regulating the gut microbiota and macrophage immunity to reshape HCC tumor microenvironment	[102]
celastrol	orthotopic HCC induced by DEN/immunocompetent model	*↓Bacteroides fragilis*	↑glycoursodeoxycholic acid	Inhibiting hepatic FXR/RXRα interaction to induce mTOR/S6K1-mediated G0/G1 arrest and suppress proliferation	[44]
xierezhuyubuxu decoction	H22 tumor-bearing Subcutaneous mice model/immunocompetent model	*↑*beneficial bacteria (such as *Akkermansia muciniphila and Parabacteroides distasonis);* *↓* harmful bacteria (*such as Barnesiella intestinihominis*)	influencing differential metabolites related to microbiota	restoring dysfunctional gut microbiota and promoting pyroptosis by NLRP3-caspase 1-GSDMD pathway	[81]
Shaoyao Ruangan mixture	Unresectable liver cancer patient/immunocompetent people	*↓Bacteroides*	NA	Inhibiting IL-10 levels	[122]
Grifola frondosa	Subcutaneous injection H22 cells/immunocompetent model	*↑norank_f__Muribaculaceae, Bacillus; ↓Lactobacillus;*	↑butyric acid	suppressing TLR4-NF-κB	[97]
Zn (II)-curcumin solid dispersion	H22 liver xenograft tumor /DEN-induced HCC model/immunocompetent model	*↑Bacteroidetes, Barnesiella, Paraprevotella, Prevotella;* *↓Firmicutes, Lachnospiraceae, Clostridium XIVa, Oscillibacter*	↑SCFAs (butyric acid)	inhibiting intestinal mucus barrier degradation, enhanced chemotherapy	[91]
Stigmasterol	Subcutaneous injection H22 cells/immunocompetent model	*↑Lactobacillus johnsonii, Lactobacillus murinus, Lactobacillus reuteri*	↑Lactobacillus metabolites	regulating Treg and IFN-γ + CD8 + cell and enhancing immune response	[106]
Taohong Siwu Decoction	subcutaneous injection of LM3 cells/immunocompetent model	*↑Duncaniella, Odoribacter, Parabacteroides, Rikenellae_RC9_gut_group, Bacteroid_unclassified, Enterococcus;* *↓Anaeroplasma, Muribacter, Aerococcus, Tannerellaceae—unclassified, Corynebacterium*	influencing differential metabolites	promoting the LTF/AMPK/mTOR/Becn1 axis and promotes lysosomal autophagy	[80]
Yinchenhao Decoction	orthotopic transplantation tumor model of HCC by H22/immunocompetent model	NA	↓deoxycholic acid, taurocholic acid, tauro β-muricholic acid, β-muricholic acid, allocholic acid and cholic acid	inhibiting the NF-κB pathway to reduce the expression of TNF-α and IL-6	[95]

### Gut microbiota modulation by TCM in HCC

In HCC, TCM bidirectionally modulates gut microbiota composition by promoting the proliferation of beneficial bacteria and inhibiting the overgrowth of pathogenic species. The core mechanisms of TCM, regardless of its formulation type—monomers, compound formulas, or extracts—center on restoring microbial diversity and optimizing community structure. For TCM monomers, berberine enriches beneficial taxa, such as *Akkermansia muciniphila* and *Lactobacillus acidophilus*, while suppressing pro-inflammatory bacteria including *Desulfovibrio* and *Bacteroides fragilis*, and enhancing microbial metabolic potential by regulating acetate kinase (ackA) activity [79]. Celastrol specifically inhibits the abundance of *Bacteroides fragilis*, reducing endotoxin release and improving the microbial colonization microenvironment [44]. TCM compound formulas also demonstrate targeted effects: Taohong Siwu Decoction (THSWD) enriches *Duncaniella*, *Odoribacter*, and *Parabacteroides* to downregulate HSC activation [80]; Xierezhuyubuxu Decoction (XRZYBXD) enriches *Akkermansia muciniphila* to block aberrant Wnt/β-catenin signaling [81]; and Sishen Wan modulates the Firmicutes/Bacteroidetes ratio to improve host-microbe crosstalk [82]. Similarly, TCM extracts, such as chlorogenic acid that inhibits *Ruminococcaceae* UCG-004 overgrowth, and Ophiopogon japonicus polysaccharides that enriches *Akkermansia muciniphila*, further restore microbial diversity and block hepatocarcinogenesis [83–85].

#### Biotransformation of TCM by gut microbiota

In the treatment of HCC with TCM, the gut microbiota-metabolism-immunity axis serves as a critical bridge linking TCM administration to antitumor efficacy, while the in vivo transformation of TCM by gut microbiota is even a core link determining therapeutic outcomes. Most TCMs for HCC treatment are administered orally and require complex absorption, transformation, and metabolism in the digestive tract to exert antitumor and immunomodulatory effects. Drug-metabolizing enzymes of the gut microbiota can catalyze hydrolysis and redox reactions of TCM components, converting inactive or low-activity ingredients into pharmacologically active compounds, reducing their polarity and molecular weight to facilitate transport to the liver (the target organ of HCC) for action, and participating in the regulation of this axis. Studies have shown that the core TCM components metabolizable by gut microbiota and involved in HCC treatment include glycosides, flavonoids, and phenolic acids, whose microbial metabolites are important substances for anti-HCC activity and immune regulation. Some glycosides only exhibit anti-HCC activity after gut microbiota transformation; for example, ginsenosides, the core components of Panax ginseng, have low oral bioavailability, but gut microbiota can metabolize ginsenosides Rb1 and Rd into compound K. This metabolite can inhibit HCC cell proliferation, arrest the G1 phase of the cell cycle, and induce apoptosis at low concentrations, which has also been verified in other tumor cells [86, 87]. After transformation by gut microbiota, flavonoids show significantly enhanced anti-HCC activity and immunomodulatory capacity. Baicalin can be rapidly converted into baicalein by the human gut microbiota, and experiments have confirmed that baicalein has much stronger tumor-inhibitory effects than baicalin, which is closely related to the regulation of the hepatic immune microenvironment [88]. Phenolic acids require gut microbiota transformation to enhance their anti-HCC activity. Fermentation of hawthorn procyanidin extract can produce short-chain fatty acids (SCFAs) to regulate gut microbiota, and its components can be converted into phenolic acid metabolites under the action of various bacteria, directly inhibiting tumors and regulating hepatic immunity through the gut-liver axis [89]. Other TCM active components related to HCC also rely on gut microbiota transformation to exert their effects. Ellagitannins in Epilobium extract, whose gut microbiota transformation product urolithins (especially urolithin B), possess tumor-inhibitory activity, providing new ideas for HCC treatment [90]. Overall, the in vivo transformation of TCM by gut microbiota is not only critical for TCM's anti-HCC effects but also a key node connecting gut microbiota, TCM metabolism, and hepatic immunity. Its metabolites can directly inhibit tumors, regulate gut microbiota and hepatic immunity, participate in the regulation of the gut microbiota-metabolism-immunity axis, and provide new theories and research directions for TCM treatment of HCC from the perspective of the gut-liver axis.

### Intestinal barrier restoration by TCM in HCC

Intestinal barrier dysfunction, also known as "leaky gut", serves as a critical link between gut microbiota dysbiosis and hepatic damage in HCC. TCM is capable to restore barrier integrity through multiple mechanisms, including the upregulation of tight junction proteins and the enhancement of mucosal barrier function. For instance, the TCM monomer Zn(II)-curcumin solid dispersion significantly upregulates the mRNA and protein expression of occludin and ZO-1 by 1.8–2.3 folds in HCC murine models, thereby strengthening intestinal mechanical barrier integrity and reducing endotoxin translocation [91]. Similarly, berberine decreases gut permeability by activating intestinal FXR signaling, which promotes tight junction remodeling in intestinal epithelial cells [85]. Moreover, TCM compound formulas demonstrates synergistic barrier-protective effects. For example, Sishen Wan improves intestinal mucosal morphology and barrier function by increasing the expression of MUC-2, occludin, and claudin, thus alleviating chemotherapy-induced barrier damage [82]. The Jianpi-Huatan-Huoxue-Anshen formula significantly enhances ZO-1 and MUC-2 expression in H22 HCC mice, mitigating gastrointestinal inflammation-mediated barrier disruption [92]. Pien Tze Huang reinforces intestinal barrier function by synergistically upregulating E-cadherin and ZO-1 expression through the enrichment of probiotics and beneficial metabolites [93]. Furthermore, TCM extracts such as Cassiae Semen extract decreases intestinal permeability in HCC models, upregulates occludin expression, and inhibits TLR4/NF-κB pathway activation induced by LPS translocation [94]. Ophiopogon japonicus polysaccharides indirectly promote MUC-2 secretion in intestinal epithelial cells by regulating gut microbiota composition, thereby reducing barrier damage-mediated hepatic inflammation [84].

### Regulation of the gut microbiota-metabolite axis by TCM in HCC

TCM modulates gut microbiota composition, thereby influencing key microbial metabolites—bile acids, SCFAs and indole derivatives—to interfere with the "microbiota -metabolite -hepatic injury" pathway. Yinchenhao Decoction has been shown to alleviate hepatic inflammation in HCC mice by regulating the bile acid profiles, reducing pro-inflammatory bile acids such as deoxycholic acid (DCA) and taurocholic acid (TCA) by 30–45% and increasing anti-inflammatory bile acids like chenodeoxycholic acid (CDCA) [95]. Similarly, Jiawei Xiaoyao San improves gut microbial diversity and modulates primary bile acid metabolism, suppressing HSC activation mediated by secondary bile acids [96]. Celastrol increases glycoursodeoxycholic acid (GUDCA) levels by inhibiting *Bacteroides fragilis*, which induces G0/G1 phase arrest in HepG2 cells [44]. SCFAs are also critical metabolites regulated by TCM. For example, berberine promotes the proliferation of SCFA-producing bacteria *Lactobacillus*, increasing intestinal acetate and butyrate concentrations [79]. This, in turn, activates peroxisome proliferator-activated receptor δ (PPARδ), upregulating apoptotic genes (BAX, cleaved Caspase-3) and downregulating anti-apoptotic BCL2, thereby inhibiting HCC cell survival [79]. In addition, Grifola frondosa polysaccharide enriches *Muribaculaceae* taxa, increasing intestinal butyrate production and suppressing the TLR4/NF-κB pathway to reduce hepatic inflammation [97]. The Zn(II)-curcumin solid dispersion modulates the Bacteroidetes/Firmicutes ratio, elevating propionate and other SCFAs to inhibit HCC growth [91]. Finally, TCM also influences indole derivative production. TCM compound formulas regulate tryptophan-metabolizing bacteria (e.g., *Bifidobacterium*, *Clostridium*), increasing intestinal indole-3-propionate (IPA) and indole-3-acetic acid (IAA) concentrations [73, 75]. Berberine promotes IPA production by gut microbiota, inhibiting hepatic inflammatory cytokine (IL-6, TNF-α) secretion and indirectly ameliorating metabolism-driven hepatocarcinogenesis [74]. In summary, TCM targets three core metabolites—bile acids, SCFAs, and indole derivatives, by regulating gut microbiota, multidimensionally regulating the pathway of "gut microbiota dysbiosis—metabolic imbalance". It not only corrects HCC-related metabolic disorders but also lays a metabolic foundation for remodeling the gut-liver-immune axis and exerting the overall anti-HCC effect.

### Remodeling of the gut-liver-immune axis by TCM in HCC

Microbial metabolites, transported to the liver via the gut-liver axis, exert significant immunomodulatory effects on hepatic immune cells, thereby establishing a sophisticated "gut-liver-immune" regulatory network. Through targeted regulation of this axis, both compound formulations and bioactive monomers derived from TCM demonstrate considerable therapeutic potential against HCC.

#### Regulation of macrophage polarization

TCM demonstrates a notable capacity to modulate the polarization states of tumor-associated macrophages (TAMs) in the liver, achieved in part through targeted remodeling of the gut microbiota. This modulation establishes a "microbiota-macrophage" axis, representing an important mechanism by which TCM influences the tumor immune microenvironment and providing a rationale for innovative strategies in HCC immunotherapy. Several lines of evidence support this mechanism. For instance, the microbial metabolite D-lactate, upon delivery to the liver via the portal vein, induces phenotypic conversion of M2 TAMs to the M1 subtype through the concurrent inhibition of both phosphatidylinositol 3-kinase (PI3K)/protein kinase B (AKT) and NF-κB signaling pathways, effectively reprogramming the immunosuppressive tumor microenvironment in HCC [98]. Similarly, compound TCM formulations have been shown to regulate the abundance of *Bacteroides fragilis* [99], which in turn promotes macrophage polarization towards the M1 phenotype, upregulates the co-stimulatory molecules CD86, and enhances innate immune responses [100]. Furthermore, Huaier polysaccharides exhibit potent immunomodulatory effects by regulating gut microbiota composition, specifically reducing *Parasutterella* while increasing *Akkermansia* populations, accompanied by decreased bile acids levels. These coordinated changes effectively suppress M2 macrophage polarization, contributing to the anti-HCC activity [101]. A representative example of TCM’s impact on gut microbiota is the combination therapy of quercetin with anti-PD-1 antibodies, which induces substantial alterations in gut microbial composition, including increased abundance of *Firmicutes*, *Actinobacteria* and *Verrucomicrobiota* at the phylum level, along with enrichment of *Dubosiella* and *Akkermansia* at the genus level. These microbial changes enhance macrophage immune function, characterized by the downregulation of M2-polarization markers including arginase-1 (Arg-1), interleukin-10 (IL-10), transforming growth factor-β (TGF-β), and matrix metalloproteinase-9 (MMP-9), and the concurrent upregulation of M1-associated genes, such as IL-12a, IL-1β, and TNF-α, alongside IL-6—a cytokine with context-dependent roles that may participate in M1-mediated anti-tumor inflammation in the HCC microenvironment[102].

#### T/NK cell function activation

Multiple studies suggest that both active compounds and complex formulations of TCM can modulate the gut microecology-metabolism axis to enhance immune responses, potentially offering novel strategies for HCC prevention and treatment. Specifically, TCM interventions can remodel the gut microbiota and regulate microbial metabolites, thereby activating antitumor T cell responses, enhancing CD4^+^ and CD8^+^ T cell populations and elevating IFN-γ levels [103, 104]. Consistent with these findings, clinical trials have confirmed that Wenzi Jiedu Recipe alters gut microbiota composition, increasing the proportion of peripheral CD8^+^ T cells and the expression of IL-10, IFN-γ, and TNF-α [105]. Notably, IFN-γ and TNF-α are key pro-inflammatory cytokines that enhance anti-tumor immunity, while IL-10 maintains immune homeostasis by suppressing excessive inflammation.

Several mechanisms underlie these immunomodulatory effects. First, TCM can directly influence the gut microbiota composition. For instance, Stigmasterol enrichs *Lactobacillus johnsonii*, *Lactobacillus murinus*, and *Lactobacillus reuteri*, reshaping the gut microbiota and upregulating lactobacillus-derived metabolites to increase the ratio of IFN-γ^+^CD8^+^ T cells to Tregs, thereby enhancing immune responses in the HCC tumor microenvironment [106]. Conversely, Safflower yellow downregulates *Alloprevotella*, *Ruminococcus*, *Barnesiella*, *Bacteroides*, and *Erysipelotrichaceae* incertae sedis in HCC-bearing mice, while simultaneously upregulating CD8^+^ T cells, promoting hepatic immune infiltration, and modulating the tumor immune microenvironment to suppress HCC progression [107]. Xiaoyuxue Decoction (XYXD) also modulates gut microbiota by upregulating *Bacteroides* and *Lactobacillus* while downregulating *Eubacterium*, leading to increased primary bile acids levels that activate NKT cells and mediate its anti-HCC effects [108]. Furthermore, TCM can upregulate intestinal *Lactobacillus* levels [109], which enhances the expression of natural cytotoxicity receptors to promote NK cell activation and innate immunity [110], and upregulate intestinal *Bifidobacterium* levels [111], which further enhances DC activation to improve tumor-specific CD8^+^ T cell responses and restore the therapeutic efficacy of anti-PD-L1 treatment [112]. Gut microbiota-derived antigens or immunomodulatory metabolites can mobilize and activate dendritic cells (DCs), reversing immune tolerance induced by immature DCs [113].

Second, TCM can modulate microbial metabolites to influence T cell function. Butyrate, a microbial metabolite regulated by TCM, directly enhances the antitumor cytotoxicity of CD8⁺ T cells by upregulating IL-12-mediated signaling dependent on DNA binding protein 2, which is supported by increased IFN-γ and granzyme B secretion in CD8⁺ T cells following butyrate supplementation [114]. It is important to note that IL-12 is a key cytokine that promotes Th1 differentiation and enhances CD8⁺ T cell effector function, including cytotoxicity and IFN-γ secretion [115]. Clinical studies have shown that Quxie capsule increases the abundance of butyrate-producing bacteria such as *Lachnospiraceae*, thereby promoting TH cell differentiation [116, 117]. These findings suggest that TCM-mediated modulation of the gut microecology-metabolism axis to enhance immune responses may serve as a potential adjuvant to improve the efficacy of PD-1/PD-L1 inhibitors.

### Clinical evidence and advantages of TCM in HCC

The clinical value of TCM in HCC treatment is fundamentally rooted in its ability to reshape the homeostasis of the "gut microbiota-metabolism-immunity axis". This "multi-link, full-chain" regulatory model, validated by numerous clinical studies, offers a novel perspective distinct from Western medicine's single-target interventions for HCC. Evidence supporting this axis regulation has become increasingly robust, demonstrating clear benefits in improving survival, preventing recurrence, and enhancing tolerability in HCC treatment. These benefits are achieved through the holistic regulation of the "gut microbiota-metabolism-immunity axis", whether using TCM compound formulations or bioactive monomers. For instance, in advanced HCC, a randomized controlled trial (RCT) of Jiedu Granule (JDG) demonstrated that the JDG group achieved a higher median overall survival (OS) than the control group. Mechanistically, JDG regulated the gut microbiota in a dose-dependent manner and reduced the relative abundance of HCC-associated pathogenic bacteria [118]. Similarly, another multi-center, open-label RCT involving 1044 patients was conducted to investigate the therapeutic impact of Huaier Granule, a TCM preparation, on HCC. These patients underwent curative resection, followed by administration of Huaier Granule as an adjuvant treatment for 6 months. The results showed that the 2-year recurrence-free survival (RFS) rate for the treatment group was 62.39%, markedly higher than that of 49.05% for control group (p = 0.0001). Additionally, Huaier Granule treatment extended the time to recurrence and reduced the recurrence rate [119]. At the mechanistic level, Huaier polysaccharides, the core bioactive component of Huaier Granule, exert their effects by regulating the "gut microbiota-M2 macrophage polarization axis", a key branch of the broader "gut microbiota-metabolism-immunity axis". Specifically, Huaier polysaccharides significantly reshapes gut microbiota composition, modulates the level of the microbial metabolite chenodeoxycholic acid (CDCA), substantially reduces M2 macrophage polarization in the tumor microenvironment (TME), and increases the secretion of pro-inflammatory factors by macrophages [101]. This cascade forms a regulatory loop of "gut microbiota-microbial metabolites-hepatic immune microenvironment", which underpins its clinical benefit in reducing post-surgical recurrence. Beyond TCM compound formulations, TCM bioactive monomers also contribute to HCC management through axis modulation. A 6-year follow-up of a RCT revealed that berberine, a well-investigated TCM monomer, possesses long-term chemopreventive potential for HCC and can effectively prevent tumor recurrence [120]. Mechanistically, berberine increases the abundance of butyrate-producing bacteria in the gut (e.g., *Lactobacillus* and *Lachnospiraceae*), thereby elevating serum butyrate levels. As a critical microbiota-derived metabolite, butyrate not only enhances intestinal barrier integrity to reduce endotoxin translocation but also modulates hepatic immune function [79]. In summary, TCM provides a novel therapeutic perspective of "starting from microecological homeostasis", breaking the limitation of Western medicine, which "only targets tumor cells or a single immune link". This opens up a new path for the comprehensive treatment of HCC and further confirms the core view of this review, the axis regulation of gut microbiota- metabolites-immune is the core innovation of TCM in HCC treatment."

## Challenges and prospects of TCM in modulating the gut microbiota-metabolism-immunity axis for HCC treatment

### Limitations and challenges

Despite the promising avenues for HCC modulation opened by research on the gut microbiota-metabolism-immunity axis, the current research paradigm encounters significant theoretical and technological obstacles that hinder clinical translation. One fundamental challenge lies in the inherent nonlinear dynamics of this hierarchical regulatory network. The gut microbiota influences the hepatic immune microenvironment through metabolites such as secondary bile acids and SCFAs, while reciprocally, the inflammatory status in the liver affects intestinal barrier integrity [123]. These bidirectional feedback mechanisms defy conventional linear causal inference frameworks. Further complicating translational efforts are several key limitations. First, the majority of clinical studies are cross-sectional, precluding the determination of whether gut dysbiosis is a cause or consequence of HCC. Second, substantial interspecies differences exist between animal models and humans, which severely impairs translational validity [124, 125]. Notably, current preclinical evidence predominantly relies on chemically induced models or diet-induced models, which exhibit inherent species-specific discrepancies with human HCC. Etiologically, human HCC is primarily driven by chronic viral infection (HBV/HCV), metabolic dysfunction-associated steatohepatitis (MASH), or alcohol-related liver disease, whereas animal models often depend on single chemical carcinogens or acute dietary manipulation, lacking the complex, long-term pathogenic cascade of human liver carcinogenesis. In terms of gut microbiota composition, the gut ecosystem of laboratory mice are dominated by Firmicutes and Bacteroidetes with low inter-individual diversity, which differs substantially from the high complexity of human gut microbiota. Metabolically, key pathways such as bile acid biotransformation and short-chain fatty acid signaling exhibit species-specific regulatory patterns. These discrepancies lead to inconsistent translatability of TCM-mediated microbiota modulation effects, as the gut microbiota-metabolite-immunity axis in animals may not fully recapitulate that in humans. Third, current omics technologies possess inherent methodological constraints: metagenomic sequencing lacks sensitivity for low-abundance taxa [126], and spatial metabolomics offers limited resolution for detailed tissue microenvironment analysis [127, 128]. Additional obstacles to clinical translation include significant individual variability in responses to microbial interventions, the absence of clearly defined synergistic mechanisms between microbiota-targeted strategies and established therapies such as immune checkpoint inhibitors, and the lack of standardized efficacy evaluation systems. Notably, the potential risks of TCM have not been systematically clarified, which increases the complexity of its clinical translation. The dynamic interaction between TCM's multi-component nature and the host's microecosystem may subtly disrupt intestinal microbial homeostasis, requiring targeted clinical monitoring; the synergistic or antagonistic effects of multiple components in TCM compound formulas on their targets may affect core therapeutic efficacy, while the underlying mechanisms remain unclear. This highlights the importance of precise dosage regulation and personalized medication, yet systematic research in this field still has gaps. There is an urgent need to improve it through multi-dimensional mechanistic exploration and clinical validation, so as to provide scientific support for the safe and effective clinical translation of TCM. Addressing these challenges necessitates an interdisciplinary research framework that integrates cutting-edge technologies such as organoid coculture systems, single-cell multi-omics, and microbiome editing, complemented by longitudinal cohort studies to elucidate temporal regulatory patterns. This comprehensive approach may facilitate a paradigm shift from correlative studies to mechanistic dissection and from population-based data to personalized therapeutic strategies.

### Prospects and opportunities

**Fig. 3:**
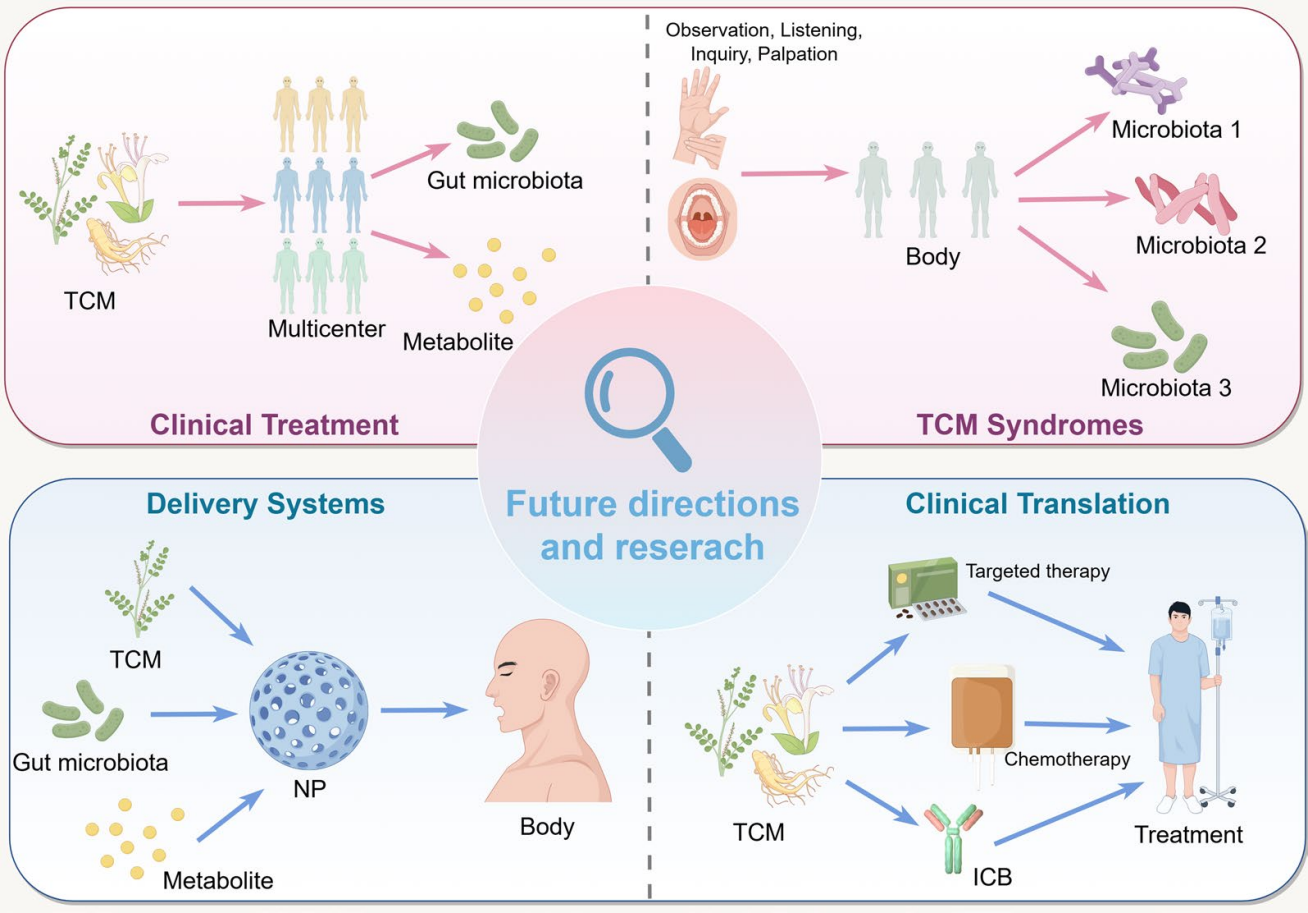
Clinical Applications and Future Research Directions of TCM in Regulating the Gut Microbiota-Metabolite-Immune Axis Network. 1. Establishing large-scale multi-center clinical cohorts is crucial to clarify the pathways through which TCM modulates the host intestinal microbiota and its metabolites. 2. Integrating modern microbiome technologies with TCM diagnostic methods - inspection, listening and smelling, inquiry, and palpation – can accurately characterize the dynamic gut microbiota profiles specific to different TCM syndrome patterns. 3. Innovative targeted nano-delivery systems can be developed by combine nanotechnology, TCM theory, and microecology. 4. Exploring multi-modal clinical translation potential is necessary, including applying TCM in targeted therapy, chemotherapy, and immune checkpoint blockade, among other intervention strategies

TCM demonstrates a unique systems medicine value in HCC treatment by regulating the gut microbiota-metabolism-immunity axis, leveraging its core advantage in multi-target, multi-level holistic regulation that reshapes the homeostasis of the gut-liver microenvironment [129]. Mechanistically, TCM modulates gut microbiota structure by promoting the proliferation of beneficial metabolite-producing bacteria while inhibiting the colonization of detrimental metabolite-producing bacteria. This, in turn, regulates the polarization of hepatic Kupffer cells and activates adaptive immunity via the "microbiota-metabolite-immune cell" axis [12, 130]. However, current translational research faces critical challenges. First, the interaction mechanisms between complex TCM components and the gut microbiota remain unclear, especially regarding microbial biotransformation of herbal compounds and their subsequent pharmacological effects. Second, large-scale cohort validation is lacking for the association between TCM syndrome patterns and specific microbiota-metabolic phenotypes. Third, traditional water decoctions encounter technical bottlenecks in microbiota-targeted delivery due to low bioavailability and imprecise targeting. Future research should prioritize several key areas. One is constructing a "TCM-microbiota-host" tripartite interaction theoretical framework by integrating cutting-edge technologies like organoid co-culture systems, spatial metabolomics, and single-cell sequencing. Another core priority is establishing gut microbiota-based TCM syndrome classification criteria, requiring individualized stratified analysis tailored to the inherent heterogeneity of HCC subtypes and TCM syndromes to construct a precision intervention framework of "syndrome differentiation-microbiota modulation-anticancer therapy." Firstly, for HCC subtype stratification, diverse etiologies (e.g., metabolic disorders, viral infections) induce distinct gut microbiota dysbiosis, and clarifying subtype-specific "microbiota-metabolite-immunity" axis alterations and their correlations with TCM syndromes establishes a solid foundation for subtype-targeted TCM interventions; secondly, for TCM syndrome stratification, HCC patients predominantly present with syndromes like Qi deficiency-blood stasis (linked to immune hypofunction and microcirculatory disturbances) and damp-heat stagnation (associated with excessive inflammation), so stratified analysis must identify syndrome-specific microbiota profiles and key metabolic mediators to develop targeted modulation strategies. Building on these, a three-dimensional "HCC subtype-TCM syndrome-microbiota phenotype" model that systematically dissects the synergistic effects of etiologies and syndromes on the gut-liver axis provides precise targets for individualized TCM formula optimization, with substantial theoretical and clinical significance. Furthermore, innovating microbiota-responsive drug delivery systems, such as pH-sensitive nanoparticles, is crucial to improve intestinal targeting efficiency [131]. In terms of clinical translation, exploring the synergistic mechanisms between TCM and immune checkpoint inhibitors is paramount, particularly the regulatory effects of microbiota remodeling on the tumor immune microenvironment in patients resistant to immune checkpoint inhibitors [132] (Fig. 3). By addressing these challenges through multidisciplinary innovation, a novel integrated HCC treatment paradigm with Chinese characteristics can be established, providing original therapeutic strategies to overcome HCC immune tolerance.

In addition, to bridge the gap between animal models and clinical translation demands prioritization of multi-dimensional optimization strategies. First, move beyond single chemically induced models to adopt human-relevant preclinical systems—such as HBV-transgenic mice fed a high-fat/high-cholesterol diet (to mimic HBV-MASH comorbid HCC) or patient-derived xenograft (PDX) models reconstituted with human gut microbiota via fecal microbiota transplantation (FMT)—to accurately recapitulate the pathogenic crosstalk between viral infection, metabolic disorders, and gut dysbiosis in humans. Second, establish humanized gut microbiota models by colonizing germ-free (GF) mice with fecal microbiota from HCC patients of distinct etiologies, to validate TCM-mediated modulation of key taxa and metabolites. Third, conduct cross-species multi-omics validation by integrating clinical and animal model data—including fecal metagenomics and serum metabolomics—to identify conserved TCM-regulated pathways while excluding species-specific non-translatable mechanisms. Fourth, construct liver-gut organoid co-culture systems, such as, 3D in vitro co-cultures of patient-derived liver and gut epithelial organoids that facilitate real-time observation of TCM effects on gut barrier function, microbial metabolite secretion, and hepatic immune cell polarization, thus circumventing species-specific microenvironmental differences. Collectively, these strategies narrow the "species gap" by aligning preclinical research with the etiological, microbial, and metabolic characteristics of human HCC, empowering findings to more reliably inform clinical trials and enhance the translational efficiency of TCM-based HCC therapies.

## Conclusion

The gut microbiota-metabolite-immunity axis in HCC represents a complex interaction network between intestinal microbial communities, their metabolic products, and the hepatic immune microenvironment, profoundly influencing HCC initiation, progression, and therapeutic response. This axis encompasses multiple core components, including gut microbiota, microbiota-derived metabolites, and diverse cellular and molecular elements within the hepatic immune landscape. Systematic investigation of regulatory mechanisms governing this axis therefore carries significant theoretical and clinical implications for developing precision microecology-targeting therapies and improving patient outcomes. Its intricate interplay critically contributes to HCC advancement, immune evasion, and treatment resistance, involving both pathophysiological processes of the gut-liver axis and immunometabolic reprogramming of the tumor microenvironment. Reflecting a deepened understanding of HCC pathogenesis, emerging treatment strategies increasingly focus on modulating the gut-liver axis and remodeling the tumor immune microenvironment, whereas conventional HCC therapies primarily target tumor cells directly. TCM exerts multifaceted effects by restructuring gut microbiota composition to promote beneficial bacteria while suppressing pathogens; enhancing intestinal barrier function to reduce endotoxin and pro-inflammatory cytokine release; and modulating hepatic immune responses via the gut-liver axis. Beyond alleviating hepatic inflammation, TCM employs an integrated "microbiota-metabolism-immunity" regulatory strategy that uniquely activates immune cells through microbiota-derived metabolites to enhance antitumor functionality. This holistic approach demonstrates multidimensional therapeutic advantages in HCC management, including direct suppression of tumor growth/metastasis, improvement of metabolic dysregulation and immune function, and ultimately enhanced quality of life and prolonged survival. TCM-based precision interventions offer novel therapeutic perspectives, particularly when combined with conventional therapies (surgery, targeted therapy, immunotherapy), where synergistic effects may provide superior clinical benefits for HCC patients. Future integration of metagenomics, metabolomics, and immunology technologies will further elucidate the mechanisms and action of TCM, establishing a robust scientific foundation for its expanded clinical application in HCC treatment.

## Data Availability

No datasets were generated or analysed during the current study.

## Abbreviations

AHR: Aryl hydrocarbon receptor
AKR1D1: Aldo–keto reductase family 1 member D1
AKT: Protein kinase B
APC: Antigen-presenting cell
Arg-1: Arginase-1
BAX: Bcl-2-associated X protein
BCL2: B-cell lymphoma 2
BCLC: Barcelona Clinic Liver Cancer
BSH: Bile salt hydrolase
CA: Cholic acid
CDCA: Chenodeoxycholic acid
CCL4: Chemokine (C–C motif) ligand 4
CREB1: CAMP response element-binding protein 1
COX-2: Cyclooxygenase-2
CXCL16: Chemokine (C-X-C motif) ligand 16
DCA: Deoxycholic acid
DC: Dendritic cell
DEN: Diethylnitrosamine
F/B ratio: Firmicutes/Bacteroidetes ratio
FXR: Farnesoid X receptor
GALT: Gut-associated lymphoid tissue
GPCRs: G protein-coupled receptors
GSDMD: Gasdermin D
GUDCA: Glycoursodeoxycholic acid
HDAC: Histone deacetylase
HDCA: Hyodeoxycholic acid
HCC: Hepatocellular carcinoma
HSC: Hepatic stellate cell
IAA: Indole-3-acetic acid
IBAT: Ileal bile acid transporter
ICB: Immune checkpoint blockade
IFN-γ: Interferon-γ
ILC3: Type 3 innate lymphoid cell
IL-1β: Interleukin-1β
IL-6: Interleukin-6
IL-10: Interleukin-10
IL-12a: Interleukin-12a
IL-17A: Interleukin-17A
IL-18: Interleukin-18
IPA: Indole-3-propionate
iso-LCA: Isolithocholic acid
JDG: Jiedu Granule
KC: Kupffer cell
LCA: Lithocholic acid
LSECs: Liver sinusoidal endothelial cells
LPS: Lipopolysaccharide
LTA: Lipoteichoic acid
MASLD: Metabolic dysfunction-associated steatotic liver disease
MAMPs: Microbial-associated molecular patterns
MMP-9: Matrix metalloproteinase-9
MUC-2: Mucin-2
mTOR: Mechanistic target of rapamycin
NFAT: Nuclear factor of activated T cells
NF-κB: Nuclear factor κB
NK: Natural killer
NKT: Natural killer T cell
NLRP3: NOD-like receptor pyrin domain-containing 3
NP: Nanoparticle
PAMPs: Pathogen-associated molecular patterns
PD-1: Programmed cell death protein 1
PD-L1: Programmed death-ligand 1
PEG2: Prostaglandin E2
PI3K: Phosphatidylinositol 3-kinase
PMCA: Plasma membrane Ca^2^⁺-ATPase
PPARδ: Peroxisome proliferator-activated receptor δ
SCFAs: Short-chain fatty acids
SASP: Senescence-associated secretory phenotype
STAT3: Signal transducer and activator of transcription 3
TAM: Tumor-associated macrophage
TCA: Taurocholic acid
TGF-β: Transforming growth factor-β
TLR2: Toll-like receptor 2
TLR4: Toll-like receptor 4
TCM: Traditional Chinese Medicine
TNF-α: Tumor necrosis factor-α
Treg: Regulatory T cell
UDCA: Ursodeoxycholic acid
ZO-1: Zonula occludens-1
